# ICU handover: are we forgetting something? A preliminary study

**DOI:** 10.1186/cc11126

**Published:** 2012-03-20

**Authors:** T Aslanidis, IL Chytas, A Kontos, I Soultati, A Efthmiou, E Geka, V Ourailoglou, E Anastasiou, M Giannakou-Peftoulidou

**Affiliations:** 1 G.H. AHEPA, Thessaloniki, Greece

## Introduction

The aim of this ongoing study is to review the process of handover in a university teaching hospital ICU, highlight areas of special interest and deficiency during the process, and improve current practice. Clinical handover, defined as a process of transferring authority and responsibility for providing care of patients from departing caregiver to named recipient, is a basic part of clinical practice. Failure to exchange essential information and focus on the important may have disastrous consequences for the patient.

## Methods

A prospective observational study was undertaken over a 22-day period to examine the quality and content of clinical handover by nightshift doctor to the medical team. Key aspects expected to be handed over included patient details, diagnosis, system - treatment domains and communication with relatives. Additional data collected also included duration of handover and frequency of interruptions.

## Results

A total of 207 sets of patients were collected during the study period. All handovers were supervised by a consultant intensivist. Clinical information handed over verbally covered reason for admission in 12% of cases, working diagnosis in 13% and current management plan in 29% (100% in these three in new admissions). Medical comorbidities where also poorly covered (8%). The handover was rather focused on special aspects of clinical information like the respiratory system (86%), fluid balance and laboratory findings (68%), infections status (67%), CNS (56%) and hemodynamics (54%), while nutrition and GI was poorly covered (20%). Only 26% of handovers covered significant changes in the last shift, 21% commented on the interventions made and 32% had a proposed plan for the forthcoming day discussed. Of the allocated 30 minutes, the duration of the handover varied from 20 to 50 minutes (average 28 minutes). There was a total of 34 interruptions over 22 days of the audited period. Reasons for interruption included telephone calls and requests from visiting teams and nurses.

## Conclusion

Our study identified that the structure of the handover was rather focused on a system-based approach. Difficulty in concentration due to fatigue or frequent interruptions prolongs its duration and disturbs the right flow of information. The senior clinician must ensure that handover should be a focused but educational experience for the trainee with appropriate feedback.
